# Logics of career choice - concept and results of an approval-sensitive career guidance workshop

**DOI:** 10.3389/fpsyg.2023.1235221

**Published:** 2024-06-21

**Authors:** Sevil Mutlu, Birgit Ziegler, Mona Granato

**Affiliations:** ^1^Institute for General Education and Vocational Education, Technical University of Darmstadt, Darmstadt, Germany; ^2^Federal Institute for Vocational Education and Training (BIBB), Bonn, Germany

**Keywords:** career choice, need for social approval, approval-sensitive-career guidance, school-to-work-transitions, vocational orientation

## Abstract

To manage the transition from school to work, young people need to learn about occupations, explore their own (professional) interests, skills and values, and make career-related decisions. According to Gottfredson’s Career Construction (CC) theory (1981), young people - influenced by their need for social approval - have already narrowed down their spectrum of acceptable occupations by the time they participate in school-based career orientation programs. Therefore, we focus on career guidance interventions that take into account their need for social approval. We present first results of an intervention study of a newly developed approval-sensitive workshop, “Logic of Career Choice.” It challenges students’ mostly unconscious need for social approval, raising awareness and prompting reflection and action on this aspect of their career choices. By analyzing a sample based on a quasi-experimental treatment-control group research design, we examine to what extent this workshop can contribute to reaching its objectives [*n* = 1236 students in secondary schools; treatment group (*n* = 766); control group (*n* = 470)]. Confirmatory factor analyses indicate the reliability of the new measurement instruments. The six examined constructs include (1) the relevance of social approval, (2) the feeling of autonomy, (3) interest in career choice, (4) reflection of needs, (5) intention to act, and (6) reported career choice activities. The results reveal partial confirmation of hypothesis (1) regarding the importance of social approval and (2) the feeling of autonomy. However, the constructs (4) reflection of needs and (5) intention to act show parallel developments in both the treatment and control groups, leading to the rejection of these hypotheses. The constructs (3) interest in career choice and (6) retrospectively reported activities regarding career choice show unexpected effects of the workshop. Interest in career choice decreases significantly more in the treatment group than in the control group, and the treatment group reports significantly more retrospective career choice activities. The discussion interprets the results in the context of their scientific and practical implications, with special attention given to the decrease in interest in career choice in the treatment group and the increased retrospective reporting of career choice activities.

## 1 Introduction

### 1.1 Approval-sensitive career orientation

Career choice is considered a developmental task of adolescence (Fend, 2001; Steinmann et al., 2018; Neuenschwander, 2020). Preparation and guidance is a central educational goal of schools (KMK, 2017). At the end of compulsory schooling, the pressure increases for students to make decisions about the continuation of their educational and professional careers for the first time. They often experience this as a critical life situation (Richardson et al., 2008). Each individual going through this process is influenced by many factors, including experiences in their social environment, their personal abilities, and their level of education (Bandura et al., 2001). A career or occupation not only provides a person with an economic livelihood, but also serves as a key to identity formation (Super, 1980). To develop this identity, the transition from school to work must be managed. This involves challenging tasks such as gathering information about different occupations, exploring one’s interests, skills and values, and making career-related decisions. Overall, career choice is a dynamic and multidimensional process that begins in childhood and becomes particularly relevant toward the end of schooling (Kracke, 2014; Driesel-Lange et al., 2020). Individualized and often intensive support is needed to successfully manage this task. Vocational guidance and counseling should make an important contribution to this (Sauermann, 2005). The form this support can take varies widely and is multifaceted. The Organization for Economic Co-operation and Development (OECD and European Commission, 2004) and a World Bank report (Watts and Fretwell, 2004) define career guidance services as services designed to help individuals of all ages and at all stages of life make decisions about education, training and employment. This definition highlights that vocational education, as an integral component of the curriculum, aims to assist specific groups of individuals in developing the skills necessary to manage their career development. There is international consensus on the definition of career guidance. It is also clear that countries around the world report the need to better prepare their students for the adult world of work, given challenges such as high technology, a global economy, and increasing social diversity. There is a general consensus that all students leaving school need to have basic skills that prepare them for the world of work (e. g., Zinser, 2003; Zahrebniuk, 2023).

In Germany, school-based career guidance programs typically commence in the seventh grade (Schröder, 2020). A key focus of these measures in Germany is the development of career choice competence (Rahn et al., 2020). These programs serve as learning opportunities designed to enhance this competence, enabling students to navigate their transition and future career paths with confidence. Career choice competence can be conceptualized as a construct that reflects an individual’s level of career orientation development. Driesel-Lange et al. (2020) understand career choice competence as “a bundle of specific cognitive abilities, motivational orientations and action skills” (p. 61). Adolescents engaging with school-based career guidance measures are at various stages in their career decision-making process; some may already have concrete career aspirations, while others might have only a vague idea of their career goals (Rahn et al., 2020). However, we assume that young people have already narrowed down their spectrum of occupational aspirations influenced by their social environment when they engage in career guidance programs at school, a process that begins in childhood and usually takes place unconsciously (Gottfredson, 2005).

These limitations relate primarily to the assessment of the social environment, the social approval of occupations, i.e., the image of occupations, which also includes gender-specific perceptions (Wicht et al., 2022). Mani and Mullin (2004) also emphasize that career choices are not solely determined by economic considerations. The desire for approval and support from members of one’s own social group influences career decisions (Phillips et al., 2001; Kracke and Noack, 2005; Bryant et al., 2006; Herzog et al., 2006). Recent research on career choice indicates that young people seek the approval of their social environment when choosing a profession and that the need for approval plays a key role in the orientation process, even if they are not aware of it. For example, a study by Matthes (2019) shows that adolescents’ propensity to choose an occupation in the nursing career field is reduced even when there is a congruence of interests, if friends or family are not expected to approve of this career aspiration. For similar results with occupations in the field of gardening see Landwehr (2020) or vocational schoolteacher (Trautmann, 2023). At the same time, little attention is usually paid to this factor in career guidance programs, as the person-environment (PE) relationship is one of the most widely used explanations of the compatibility between people and their work environment (Kristof, 1996; Vleugels et al., 2023).

Since the image of occupations represents a complex of subject-bound evaluations influencing occupational decisions, there is a need for research on vocational training measures that consider the need for social approval (Ertl, 2023). This paper introduces the initial findings of a recently developed career guidance program that is sensitive to the aspects of approval, based on an intervention study. Initially, the theoretical foundation supporting the intervention is presented. Subsequently, there is an elucidation of the intervention itself along with its underlying objectives. In the fourth chapter, the methodology section outlines the research design, sample characteristics, and survey instruments before introducing the hypotheses. The result section focuses on the comparison between the treatment and control groups on the constructs of interest here: *relevance of social approval*, *feeling of autonomy*, *interest in career choice*, *reflection of needs* and *intentions to act* as well as *retrospectively reported career choice activities*.

## 2 Theoretical foundation of the concept of approval-sensitive vocational orientation

It is undisputed that human beings are considered social beings who are fundamentally dependent on social interactions and community for their physical and psychosocial development (Bischof-Köhler, 2011). According to Mead (1934), self-perception and self-understanding develop through role-playing and form the basis for symbolic interactions. The concept of the “reflected or mirror self” can already be found in Cooley (1902, p. 152). Conceptions of the self are, in a sense constituted by individuals recognizing themselves, in the “social mirror” (Stets and Burke, 2003). Moreover, the development of initial concepts about the self and the social environment in infancy also begins through mere observation of other people (Bischof-Köhler, 2011). In this process, concepts as work and occupation become part of children’s imaginations. This is not surprising, considering that children often perceive adults in various occupational roles, weather real or through media, and they also learn about parental occupations through their parents’ availability. For instance, Levy et al. (2000) discovered that 3-year-old children can already distinguish demonstrated occupations into female and male occupations. Gottfredson (1981) refers to the fact of early development of occupational preferences via assessment processes of self-concept and occupational concepts in Circumscription and Compromise Theory (CC-theory). She insinuates the delimitation of an individual occupational aspiration field over four stages in which successively certain aspects of occupations become salient in addition to already existing ones and corresponding processes of exclusion take place. In the first stage, from about the age of three, there is an “orientation to size and power” (p. 548); in the second stage, from about the age of six, there is an “orientation to sex roles”; in the third stage, from about the age of nine, there is an “orientation to social valuation”; and finally, in the fourth stage, adolescence, there is a stronger “orientation to the internal, unique self” (p. 549). Gottfredson bases her theory on five developmental principles: 1) Progressive cognitive development from the concrete to the abstract. 2) Largely unconscious assessments between the self-concept and occupational concepts by intuitively matching characteristics of occupations with the self. 3) Overlapping differentiation and integration stages, especially at stage transitions, according to which, for example, children already perceive gender differences between occupations in the first stage but do not form a stable gender identity until the second stage. 4) A progressive and irreversible elimination of occupational alternatives, which has a functional advantage because this relieves cognitive and emotional strain. 5) The ability to spontaneously eliminate occupations, without being able to spontaneously verbalize one’s own preferences (Gottfredson, 1981). Empirically, the developmental model is largely confirmed in its core elements. Partly deviating findings are reported regarding the age information on the stages and the importance of interest for career choice (Ratschinski, 2009) which, however, were also largely anticipated by Gottfredson (Ziegler, 2019). For the career choice process, it can be concluded that many occupations were already excluded from the aspiration field in the phase of diffuse career orientation based on mostly stereotypical occupational concepts and largely unconsciously and sustainably. In CC-theory, Gottfredson describes internal processes that are predominantly based on the identification potential that individuals perceive in occupations for their self-concept and are increasingly evaluated in terms of their accessibility (Gottfredson, 1981). What the theory does not explicitly integrate is adolescents’ dependence on reactions from their social environment.

Therefore, going beyond CC-theory, the concept of approval-sensitive career guidance assumes that adolescents anticipate reactions of their social environment to career aspirations and also engage in “impression management” (Goffman, 1969; Mummendey, 2002) in the career choice process by excluding occupations from the aspirational field to which they do not expect a positive reaction (Oeynhausen and Ulrich, 2020). Fundamental to this is the need to develop a positive image of oneself (self-enhancement) and to receive confirmation for this (self-verification) (Leary, 2007). Impression management probably influences efforts to concretize career aspirations, especially in the psychosocially sensitive phase of adolescence. The need for social approval is not a primary concern (Baumeister, 1995; Bargh and Chartrand, 1999; Dweck, 2006; among others), but is significant for the development of social identity (Baumeister, 1995). Occupations contribute greatly to the development of social identity (Gildemeister and Robert, 1987, p. 73; Heinrichs et al., 2022). Therefore, it is hypothesized that vocational exploration behavior and decisions in the transition process are also influenced by the adolescents’ need for approval without being fully aware of it. Based on this, a workshop was designed for the intervention study in which adolescents are explicitly confronted with their need for social approval and the possible premature exclusion of occupations from their aspiration field (Oeynhausen and Mutlu, 2022).

## 3 Design of an approval-sensitive workshop: design and research questions

### 3.1 Design of an approval sensitive workshop

The intervention was designed for use in secondary schools beginning in the seventh grade. The workshop aims to empower young people to take a reflective view of their career decision-making. In some ways this is a risky endeavor, as young people facing their first career decision may tend to other choice goals or may be looking for certainty rather than deliberation. However, we assume that reflecting on one’s own preferences and choice goals when assessing career alternatives is significant for mature career decisions (Sauermann, 2005). The 90-min logic of career choice workshop uses playful and discursive interactions to explicitly address the need for approval (see Table 1). Young people are challenged in their previous perceptions of occupations while being encouraged to reflect on previously discarded career choices, to consider social categories of occupational perceptions, especially gender and prestige, and to reconsider their individual criteria for career choice. Based on the concept of reframing (Esser, 2020), this is intended to trigger new thought processes and help young people identify and question their previous motives for career choices (Oeynhausen and Mutlu, 2022). The upcoming section introduces the derived research questions.

**TABLE 1:** Workshop flow chart.

Phase	Content	Description	Aim
1	Introduction	The workshop begins with personal insights into the career paths of the workshop leaders and a short game to get to know each other.	The game engages students, offering insights into their current career choice stage. They realize they’ve unconsciously dismissed certain careers, limiting their future options
2	Career Choice Test	Students take a career test, evaluate interests and strengths, sort suggested careers using cards and pictures, and state reasons for excluding options.	The students are engaging with the logic of career choice tests in general. The basis for such tests is the classic RIASEC model by Holland (1973)^1985^. Other influencing factors, such as occupational conditions or the profession images, are typically not taken into account in these tests
3	Career Choice Theory	The workshop leader introduces Gottfredson’s theory, highlighting how children unconsciously exclude gender-mismatched careers in elementary school and, as teenagers, tend to exclude occupations with “low” prestige based on their social status.	Gottfredson’s theory familiarizes individuals with potential unconscious exclusion factors. Through the reconstruction of a “cognitive map of occupations” conscious reflection becomes accessible (Gottfredson and Lapan, 1997; Ziegler et al., 2020).
4	Need for social approval for Career Choice	The workshop leader summarizes that a central mechanism behind the exclusion of occupations due to lack of gender and prestige adequacy lies in the need for approval.	The goal of this summary is to engage in an open conversation with the adolescents about their stance on this thesis and how significant the need for social approval was and is for them in the context of career choice.

Detailed overview in Oeynhausen and Mutlu (2022).

### 3.2 Research questions

The aim of the workshop is to enhance self-knowledge and personal responsibility by encouraging adolescents to become aware of the reasons for excluding certain occupations. This process also challenges the feeling of autonomy and prompts discussions on the extent to which career decisions are genuinely autonomous. The goal is to strengthen the decision-making abilities of young individuals through this conscious examination, motivating them to engage more deeply with their career choices. Additionally, the workshop aims to activate students, initiating at least some level of concretizing their career aspirations. Focusing on a specific career goal enhances the likelihood of successfully navigating the transition process (Rahn et al., 2014). A career aspiration serves as a “catalyst for exploration and planning processes” (Driesel-Lange et al., 2023, p. 102).

Self-knowledge is recorded by asking the young people how important the approval of parents and friends is to them when choosing a career, or how autonomously they feel in making their career decisions. Regarding to further factors, the study refers to the dimensions of an occupational competence model (Driesel-Lange, 2012) and records “*interest in career choice*” as a motivational component, “*reflection of needs*” as an additional cognitive component and “*intention to act*” as an action component as well as retrospectively reported activity (*retrospective career choice activities*). The extent to which the need for approval influences vocational competence has not yet been investigated. This raises the question of whether the reflection of (unconscious) approval needs in the career choice process can activate young people cognitively and motivationally and thus contribute to an increase in their vocational competence. All constructs were reformulated for the study. In a first step, the factorial structure of the constructs is therefore examined (Ch. 4.3). In a second step, overall analyses on the effects of the intervention study on the variables mentioned above are presented (Ch. 5). The following research questions will be addressed:

1. How does the workshop affect the meaning of approval by others and the feeling of autonomy?
2. Which impact does the workshop have on the motivational, cognitive and action-related dimensions of career choice competence?
3. Does the workshop have an activating effect and does it promote career choice-related activities among the young people?

## 4 Materials and methods

### 4.1 Research design

**FIGURE 1:**
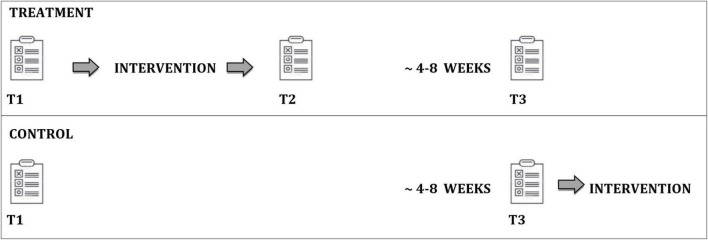
Research design. © BIBB-TUDa-Berufsorientierungsstudie.

The intervention study on the “Logics of Career Choice” workshop was conducted in the school years 2021/22 and 2022/23 in a quasi-experimental treatment- control group design. Surveys in the treatment group took place immediately before the workshop (T1), immediately after (T2), and as a follow-up (T3) after approximately 4–8 weeks. For capacity reasons, no comparable intervention could be implemented in the control group, which were therefore only surveyed at T1 and T3 and received an intervention after T3 (see Figure 1). The survey was conducted using a standardized online questionnaire on site in the classes. Some of the workshops were conducted by the first author herself as well as by six specially trained workshop leaders.

The selection of participating classes was planned as a lumped sample. The schools were divided into clumps according to survey regions and different school types. For reasons of research economy, purposive distributions were made. Treatment and control classes were interviewed at different schools whenever possible.

### 4.2 Sample

The analyses reported here are based on a sample of *n* = 1236 students, including the treatment group with *n* = 766 and the control group with *n* = 470 students. The treatment sample includes only cases that participated in the workshop at all three measurements of T1, T2, and T3 and allowed 100% agreement in the assignment of the survey time points. For the control group, data are available at measurement time points T1 and T3. The assignment could be performed anonymously using an especially for this purpose elaborated code (Schratz, 2020). According to the German school system, there is a division of students into school types with different grades. Most of them are represented in the sample. Upper secondary school leading to university entrance (Gymnasium) 41%, junior high school leading to the intermediate school certificate (Realschule) 38%, and secondary schools offering basic secondary education (19.5% Hauptschule & 2.5% Gesamtschule). In the treatment group, 13.2% of students are in grade 8, 54.8% are in grade 9, 24.1% are in grade 10, and 8% of students are in grades 11 and 12. In the control group, 9.1% attended the 8th grade level, 67.2% attended the 9th grade level and 10th grade level amounts to 23.6% of students. The gender ratio was roughly balanced, with 48.9% female and 49.8% male adolescents. Just under 1.2% classified themselves as diverse. The data collection took place until the end of the 2022/23 school year.

### 4.3 Measurement instruments

Reflecting on the need for approval in career choice has yet not been a specific objective of career counseling programs. Nevertheless, a survey instrument was pragmatically employed to operationalize the constructs. This instrument was derived from an intervention study in which the effects of different methods for assessing the vocational interests and abilities of young people were analyzed within the same target group “Potenzialanalyse” (“potential analysis”) (Sommer and Rennert, 2020). Relevant items were meticulously selected and adjusted to align with the goals of this study, particularly in evaluating self-reflection and intentions to act.

Self-perception is assessed through questions about the importance of parental and peer approval (*relevance of social approval*) and perception of autonomy in career decisions. The latter is captured under the term *feeling of autonomy*. Since parents significantly influence their children’s socialization, which in turn impacts their vocational orientation and choices (Bryant et al., 2006), their role is crucial. Many adolescents consider their parents the primary and most significant source of guidance regarding career decisions (Bryant et al., 2006; Dombrowski, 2015; Boockmann et al., 2017; Kazi and Akhlaq, 2017). The personal environment of adolescents includes not only parents but also friends or peer groups. Sinclair et al. (2018) demonstrated in their study that friends can influence educational decisions for adolescents. But there is also a need for autonomous action, as career choice is a developmental task of adolescence perceived by children as a task they want to manage independently (Havighurst, 1973; Baumann and Kuhl, 2005; Fend, 2005). With regard to the relevant dimensions of career choice competence (Driesel-Lange, 2012), the importance of approval from parents and peers and the self-assessment of autonomy as well as the scale for the *reflection of needs*, which measures how important it is for young people to consider their own needs when choosing a career, relate to the cognitive component of career choice competence. As a motivational component of career choice competence, the *interest in career choice* scale was employed to gauge young people’s importance in dealing with career choices, occupations, and their interests and abilities. The readiness to transition into action mode in the career choice process, as an action component, was assessed through the *intention to act* scale. This scale was taken from the study by Sommer and Rennert (2020), albeit with fewer items. The scales *interest in career choice* and *reflection of needs*, on the other hand, were a compilation of items from different scales (“concern”, “emotion/motivation for career choice” and “reflection”) of the same instrument. These items were linguistically adapted and augmented (see Table 2).

**TABLE 2:** Construct and item overview. © BIBB-TUDa-Berufsorientierungsstudie.

Construct	Items
relevance of social approval	**It is important for me, …** … that my parents also like a profession … that my friends also like a profession
feeling of autonomy	**I don’t care what others think of my profession - as long as I’m happy with it!**
interest in career choice	**It is important for me, …** … to deal with my career choice … to find out which professions fit my interests … to find out which professions fit my strengths … to deal with what I am good at and what I am not so good at
reflection of needs	**It is important for me, …** … to find out what exactly I like about a profession … to clarify what is important to me about my profession later on … to find out which professions fit what is important to me in life … to consider what it means to me to earn a lot of money … to consider whether a profession in which mainly men work is also an option for me … to consider whether a profession in which mainly women work is also an option for me … to consider how much I want to base my career choice on other people’s opinions … to clarify in which environment, I would like to work (e.g., in nature, in a workshop, in an office, rather alone or among people)
intention to act	**As for your career choice, what will you be doing soon?** I will inform myself intensively about different professions I will ask several people specifically for information about careers and training opportunities I plan to ask other people where they see my strengths and weaknesses I plan to take a career choice test and think about the suggested careers
retrospective career choice activities	**Since our last survey…** … I have been thinking a lot about my career choice … I have been thinking about what is most important to me in my future profession … I’ve been thinking about how important it is to me that others think my profession is good … I’ve been thinking about how important it is for me to choose a gender-typical profession … I have talked to my parents about my career choice … I talked to my friends about my career choice … we have had other career orientation events at school that have helped me a lot

A five-point Likert scale (1 = strongly disagree, 2 = disagree, 3 = neither agree nor disagree, 4 = agree, and 5 = strongly agree) was provided for all scales.

Both the workshop and the questionnaire underwent several times qualitative pretesting. The questionnaire pretest primarily focused on assessing the linguistic comprehension of item phrasing and response times, and these items were thoroughly discussed with the students. Given the diverse range of schools and grade levels surveyed, this aspect held significant importance. Pretest results indicated minor adjustments in the questionnaire wording.

**FIGURE 2:**
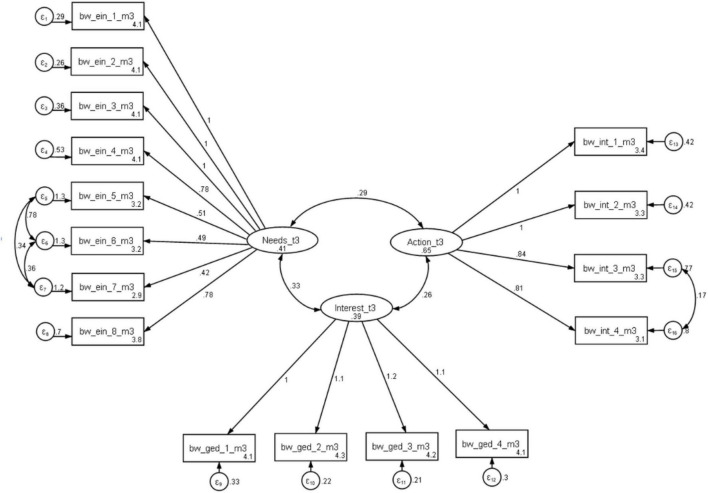
Three-factor model at T3. Variables bw_ein_1_m3 to bw_ein_8_m3 are Items for the Construct reflection of needs of T3. Variables bw_ged_1_m3 to bw_ged_4_m3 are Items for the Construct interest in career choice of T3. Variables bw_int_1_m3 to bw_int_4_m3 are Items for the Construct intention to act of T3. © BIBB-TUDa-Berufsorientierungsstudie.

The hypothetical 3-factor overall model of career choice competence is tested across all three measurement points, while the construct of retrospective activities is only examined at the third measurement point. However, both are assessed for their overall model quality using confirmatory factor analysis. The data from the treatment and control group were combined for the model test. The quality of the model is evaluated following the recommendations of Schermelleh-Engel et al. (2003), considering various criteria for assessing the quality of structural equation models. Kline (2015) recommends using indices such as the Comparative Fit Index (CFI), the Tucker-Lewis Index (TLI), and the Root Mean Square Error of Approximation (RMSEA) for data analysis in structural equation modeling (SEM) in Stata 17. These indices serve as quantitative measures to assess the degree to which the specified model aligns with the observed data. The internal consistencies of the scales were in the acceptable to good range. The quality and structure of the instrument for recording professional competence were confirmed across all three measurement points. The expected factorial structure of the test, capturing the three dependent variables, remained consistent for T1, T2, and T3. Covariances between items were considered to improve model fit. Figure 2 depicts the 3-factor model at T3.

Model analysis is presented in Table 3, displaying fit values for all three measurements. The cutoff range for good to moderate fit was between 0.05 and 0.08 for the RMSEA calculation (Browne and Cudeck, 1989; Hu and Bentler, 1998). A good model fit required a CFI value of at least 0.95 (Hu and Bentler, 1998), while a TLI value above.95 was considered acceptable (Schumacker and Lomax, 2004). The model fits indicated a satisfactory to good range of values for all measurements of the three-factor model. All chi-squared values fell within the satisfactory range, and values for RMSEA (.05), TLI (.90), and CFI (.95) were met (see Table 3).

**TABLE 3:** Goodness of fit of the three-factor model at T1, T2, T3. © BIBB-TUDa-Berufsorientierungsstudie.

Model	^χ^ ^2^	Df	CFI	RMSEA	TLI	AIC
1. Measurement	353.007	93	0.97	0.04	0.96	74519.86
2. Measurement	482.445	96	0.96	0.05	0.95	51968.69
3. Measurement	485.355	97	0.96	0.05	0.95	57792.09

CFI, Comparative-Fit-Index; RMSEA, Root-Mean-Square-Error of Approximation; AIC, Akaike’s information criterion; TLI, Tucker-Lewis Index.

**FIGURE 3:**
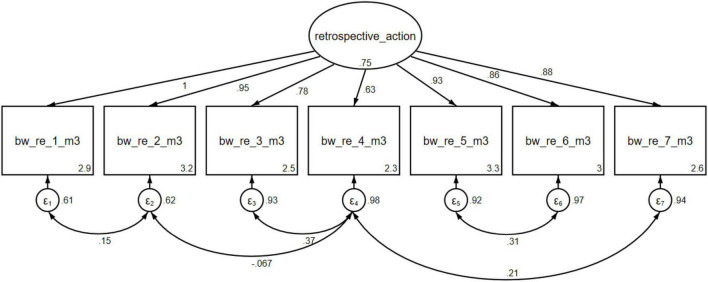
Construct: retrospective career choice activities. Variables bw_re_1_m3 to bw_re_7_m3 are Items for the Construct retrospective career choice activities of T3. © BIBB-TUDa-Berufsorientierungsstudie.

To examine whether a temporal distance provides students with the opportunity to reflect on their impressions and gain deeper insights, the third survey instrument (T3) included the scale *retrospective career choice activities* (see Figure 3). The items were developed specifically for this purpose and were examined for their consistency through confirmatory factor analysis. The quality and structure of the *retrospective career choice activities* instrument were also examined and demonstrated homogeneity, with all items loading onto one factor. Validation was performed by incorporating both treatment and control groups.

The scale *retrospective career choice activities* exhibited high internal consistency (α = 0.83). Again, covariances between items were allowed to improve model fit. The model fits showed a satisfactory to good range of values for the construct. The RMSEA value fell within the satisfactory range (.05). The cutoff value for a good to satisfactory fit was.05 for the RMSEA calculation, ranging from 0.05 to 0.08 (Browne and Cudeck, 1989; Hu and Bentler, 1998). The CFI value of.98 met the criteria for a good model fit (Hu and Bentler, 1998), and the TLI value of.96 was also acceptable (Schumacker and Lomax, 2004).

The model was able to confirm the expected three-factorial structure as well as the construct for capturing *retrospective career choice activities*. In the following chapter, the hypotheses will be explained and subsequently tested.

### 4.4 Hypotheses

Treatment effects are expected on the scales *relevance of social approval* and *feeling of autonomy*, as well as *interest in career choice*, *reflection of needs*, and *intention to act* as subcomponents of career choice competence. It is anticipated that awareness of the need for social approval will increase while the feeling of autonomy decreases, given that these aspects are extensively addressed in the intervention. Young individuals are confronted with the realization that their considerations and decisions regarding career choices may have been influenced by external factors, leading to less self-determined choices. An increase in the willingness to reflect on one’s own needs should contribute to an increase in self-knowledge as a cognitive dimension of career choice competence. Regarding the scale *interest in career choice*, however, effects in both directions are conceivable: on the one hand, an increase in interest, which would be positive as an intervention outcome. On the other hand, interest in *career choice* could also decrease due to the intended irritation in the workshop. The adolescents are unsettled in their perception of autonomy and competence, and thus, two basic needs (Deci and Ryan, 1985), which are conditions for an increase in interest, are not fulfilled (Krapp, 2002). Analogously, positive as well as negative effects are conceivable with regard to *intention to act*, an increase in interest should have a positive effect on *intention to act* and vice versa according to interest theory (Krapp, 2002). In addition, the young people were asked in the last testing (T3) which career choice-related activities they had undertaken since the last survey (scale: *retrospective career choice activities*). The hypotheses (H1-H6) pertain to the impact of the intervention, drawing comparisons between the treatment and control groups. Based on the aforementioned explanations, the following hypotheses (H) are formulated:

*H1: The relevance of social approval in career choice increases more in the treatment group than in the control group.*

*H2: The feeling of autonomy decreases more in the treatment group than in the control group.*

*H3: The intervention influences interest in career choice more in the treatment group than in the control group.*

*H4: The intervention influences reflection of needs more in the treatment group than in the control group.*

*H5: The intervention influences the intention to act more in the treatment group than in the control group.*

*H6: The treatment group retrospectively reports more career choice activities than the control group.*

In accordance with the theoretical framework and formulated hypotheses presented, the data will be examined using descriptive analyses. To determine the effects of the intervention on the dimensions of career choice competence, analysis of variance with repeated measurement and group factor are used (mixed ANOVA/ repeated measurement ANOVA). Here, the repeated measurement factor (pre- and post) represents the within-subject factor and the group factor determines membership in the treatment or control group (between-subject factor). Statements about the strengths of the treatment effects are reported descriptively when the results are presented. Further scale characteristics such as scale means and standard deviations are calculated on the basis of classical test theory using SPSS Statistics software (version 27) and presented with the respective structural tests.

## 5 Results

First, the descriptive results are presented, followed by the testing of hypotheses (Table 4).

**TABLE 4:** Descriptive statistics. © BIBB-TUDa-Berufsorientierungsstudie.

Scale (number of items)	t1			t2			t3		
	***N***	***M***	**SD**	***N***	***M***	**SD**	***N***	***M***	**SD**
**Treatment**									
Relevance of social approval (2)	763	2.25	0.94	756	2.47	0.98	747	2.39	0.89
Feeling of autonomy (1)	763	4.34	0.88	755	4.06	1.07	747	4.11	1.01
Interest in career choice (4)	764	4.32	0.61	756	4.08	0.75	750	4.13	0.76
Reflection of needs (8)	764	3.78	0.55	756	3.68	0.63	747	3.64	0.64
Intention to act (4)	760	3.35	0.82	751	3.39	0.82	745	3.24	0.83
**Control**									
Relevance of social approval (2)	456	2.26	0.93				468	2.35	0.92
Feeling of autonomy (1)	456	4.24	0.96				468	4.09	1.03
Interest in career choice (4)	459	4.35	0.57				468	4.26	0.68
Reflection of needs (8)	458	3.78	0.54				468	3.71	0.59
Intention to act (4)	454	3.36	0.78				466	3.28	0.84

*M*, Mean; SD, standard division.

Reliabilities were calculated for all scales and measurements and show satisfactory to good values ranging from .71 to .89.

**FIGURE 4:**
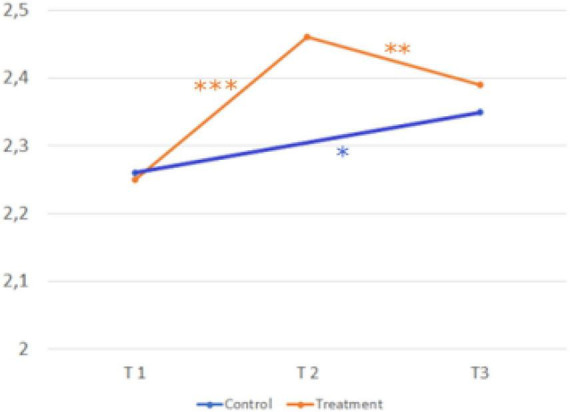
Development over time and comparison of the two groups (relevance of social approval). Sample Item*:* It is important to me that my parents/friends also like a profession. A five-point Likert scale (1 = strongly disagree, 2 = disagree, 3 = neither agree nor disagree, 4 = agree, and 5 = strongly agree) was provided for all scales. © BIBB-TUDa-Berufsorientierungsstudie.

**FIGURE 5:**
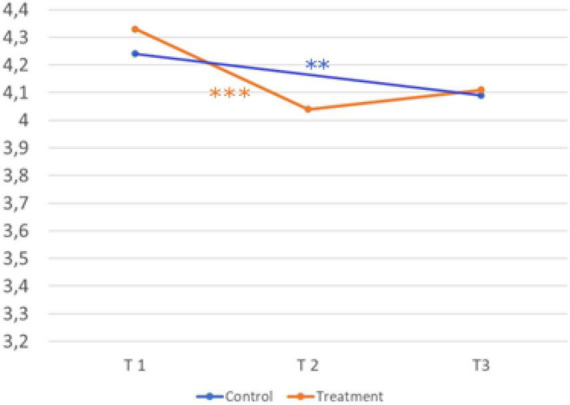
Development over time and comparison of the two groups (feeling of autonomy). Item: I don’t care what others think of my profession - as long as I’m happy with it! A five-point Likert scale (1 = strongly disagree, 2 = disagree, 3 = neither agree nor disagree, 4 = agree, and 5 = strongly agree) was provided for all scales. © BIBB-TUDa-Berufsorientierungsstudie.

Now follows the testing of hypotheses H1 - H6. H1 to H5 were conducted by modeling the respective interaction effect “measurement time” x “group” by mixed ANOVAS with measurement repetition for the constructs. For hypothesis 6, an independent samples *t*-test was employed. Hypotheses 1 and 2 refer to the need for approval by one’s own social environment. Figures 4, 5 show the development over time and the comparison of the treatment and control group.

*H1: The relevance of social approval in career choice increases more in the treatment group than in the control group.*

Scores in the treatment group (T1, *M* = 2.25; *SD* = 0.94) → T2 *M* = 2.47; *SD* = 0.98; *d* = 0.29) were significantly higher after the second measurement t(752) = −8.179, *p* < .001 and significantly lower after the third measurement (T2, *M* = 2.47; *SD* = 0.98) → T3, *M* = 2.39; *SD* = 0.89; *d* = 0.07), t(737) = 2.034, *p* < .005. Scores in the control group were significantly higher after the third measurement, t(453) = −2.156, *p* = 0.032, (T1, *M* = 2.26; *SD* = 0.93) → T3, *M* = 2.35; *SD* = 0.92; *d = 0.1*). There was no significant interaction between time and group for the construct relevance of social approval, *F*(1,1235) = 1.793, *p* = 0.181. Despite the within-subject effects in the treatment and control group, hypothesis one cannot be confirmed.

*H2: The feeling of autonomy decreases more in the treatment group than in the control group.*

Scores in the treatment group (T1, *M* = 4.34; *SD* = 0.88 → T2, *M* = 4.06; *SD* = 1.07; *d* = 0.26) were significantly lower after the second measurement t(751) = 7.109, *p* < 0.001. Scores in the control group (T1, *M* = 4.24; *SD* = 0.96 → T3, *M* = 4.09; *SD* = 1.03, *d* = 0.13) were significantly lower after the third measurement, t(453) = 2.833, *p* = 0.005. There was no significant interaction between time and group for the construct feeling of autonomy, *F*(1,1235) = 1.729, *p* = 189. Despite the within-subject effects in the treatment and control group, hypothesis two cannot be confirmed.

**FIGURE 6:**
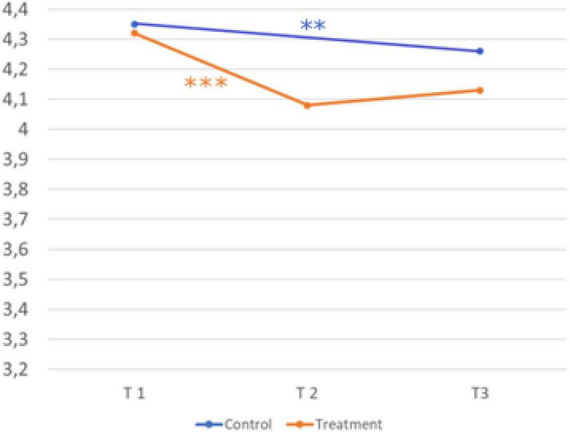
Development over time and comparison of the two groups (interest in career choice). Sample Item: It is important for me, to deal with my career choice. A five-point Likert scale (1 = strongly disagree, 2 = disagree, 3 = neither agree nor disagree, 4 = agree, and 5 = strongly agree) was provided for all scales. © BIBB-TUDa-Berufsorientierungsstudie.

The third hypothesis shows treatment effects on the motivational component (*interest in career choice*). Figure 6 shows the development over time and the comparison of the treatment and control group.

*H3: The intervention influences interest in career choice more in the treatment group than in the control group.*

Scores in the treatment group (T1, *M* = 4.32; *SD* = 0.61 → T2, *M* = 4,08; *SD* = 0.75; *d* = 0.37) were significantly lower after the second measurement t(753) = 10.394, *p* < .001. Scores in the control group (T1, *M* = 4.35; *SD* = 0.57 → T3, *M* = 4.26; *SD* = 0.68, *d* = 0.13) were significantly lower after the third measurement, t(456) = 2.862, *p* = 0.004. The construct interest in career choice, shows a significant interaction between time and group *F*(1,1242) = 7.03, *p* = 0.008, $\eta_{p}^{2}$ 0.006. So, hypothesis three receives confirmation.

**FIGURE 7:**
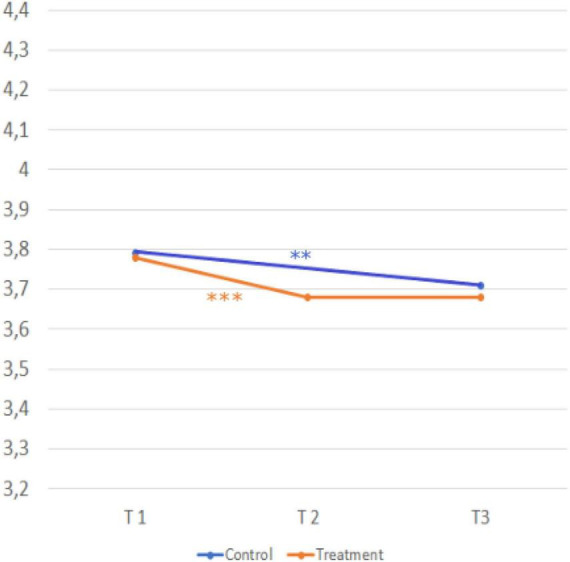
Development over time and comparison of the two groups (reflection of needs). Sample Item: It is important for me to find out what exactly I like about a profession. A five-point Likert scale (1 = strongly disagree, 2 = disagree, 3 = neither agree nor disagree, 4 = agree, and 5 = strongly agree) was provided for all scales. © BIBB-TUDa-Berufsorientierungsstudie.

The fourth hypothesis assumes an influence of the intervention on the reflection of needs. Figure 7 shows the development over time and the comparison of the treatment and control group.

*H4: The intervention influences reflection of needs more in the treatment group than in the control group.*

Scores in the treatment group (T1, *M* = 3.78; *SD* = 0.55 → T2, *M* = 3.68; *SD* = 0.63; *d* = 0.15) were significantly lower after the second measurement t(753) = 4.039, *p* < .001. Scores in the control group (T1, *M* = 3.78; *SD* = 0.54 → T3, *M* = 3.71; *SD* = 0.59, *d* = 0.13) were significantly lower after the third measurement, t(455) = 2.837, *p* = 0.005. There was no significant interaction between time and group for the construct reflection of needs, *F*(1,1238) = 1.004, *p* = 0.426. Hypothesis four cannot be confirmed.

**FIGURE 8:**
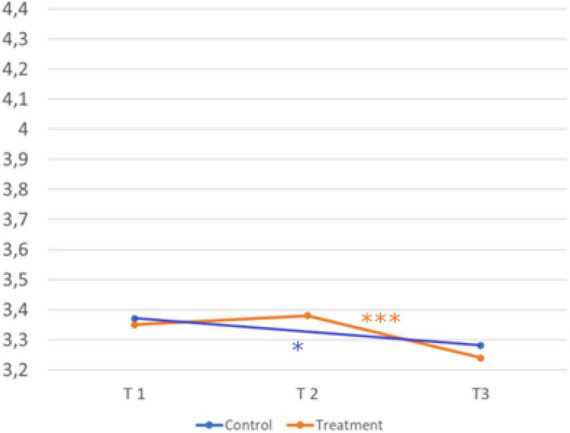
Development over time and comparison of the two groups (intention to act). Sample Item: I will ask several people specifically for information about careers and training opportunities. A five-point Likert scale (1 = strongly disagree, 2 = disagree, 3 = neither agree nor disagree, 4 = agree, and 5 = strongly agree) was provided for all scales. © BIBB-TUDa-Berufsorientierungsstudie.

Hypothesis five assumes an effect of the intervention on intention to act. Figure 8 shows the development over time and comparison of the treatment and control group.

*H5: The intervention influences the intention to act more in the treatment group than in the control group.*

Scores in the treatment group (T2, *M* = 3.39; *SD* = 0.82 → T3, *M* = 3.24; *SD* = 0.83; *d* = 0.20) were significantly lower after the third measurement t(730) = 5.433, *p* < 0.001. Scores in the control group (T1, *M* = 3.36; *SD* = 0.78 → T3, *M* = 3.28; *SD* = 0.84, *d* = 0.13) were significantly lower after the third measurement, t(450) = 2.203, *p* = 0.028. There was no significant interaction between time and group for the construct intention to act, *F*(1,1226) = 0.575, *p* = 0.45. Hypothesis five cannot be confirmed.

**FIGURE 9:**
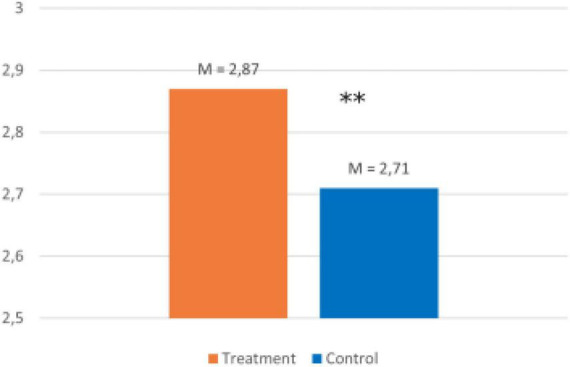
Comparison of the two groups (retrospective career choice activities T3). Sample Item: Since our last survey, I have been thinking a lot about my career choices. A five-point Likert scale (1 = strongly disagree, 2 = disagree, 3 = neither agree nor disagree, 4 = agree, and 5 = strongly agree) was provided for all scales. © BIBB-TUDa-Berufsorientierungsstudie.

The sixth hypothesis assumes higher activation to career choice as a result of the intervention. Figure 9 shows the comparison of the treatment and control group.

*H6: The treatment group retrospectively reports more career choice activities than the control group.*

Since retrospective actions were only collected in the follow-up survey, a Welch test is used to test the hypothesis. This proves to be robust regardless of variance homogeneity (Kubinger et al., 2009).

In the control group (*n* = 461), the mean is *M* = 2.71 (*SD* = 0.89); in the treatment group (*n* = 742), the mean is *M* = 2.87 (*SD* = 0.80). There is a significant difference between the groups t(893,13) = 3.11, *p* = 0.002, *d* = 0.14. Students from the treatment group retrospectively report more career choice related activities. Thus, hypothesis six can be confirmed.

Hypotheses 1 and 2 refer to the awareness of the need for approval in one’s own social environment. The results confirm these expectations but only within group and no interaction between time and group. So, both hypotheses have to be rejected. Interest in career choice decreases more in the treatment group than in the control group (H3). At the third time point, the treatment group reports more activities relevant to career choice than the control group (H6). There are no treatment effects regarding the reflection of needs and intentions to act (H 4, H5). Both groups show largely parallel and decreasing mean values. Thus, only hypotheses 3 and 6 are supported.

## 6 Discussion

The aim of the workshop is to raise awareness of needs that are sometimes unconscious. Young people are expected to make reflective decisions and actively include these needs in their decision-making process. This process entails the outcomes of H1 and H2. Immediately after the intervention, there was a significant increase in the *relevance of social approval* and a significant decrease in the *feeling of autonomy*. As assumed in hypotheses the workshop also triggered an irritation reaction, as the admission to be dependent on the opinions of others could be (first) detrimental to self-esteem and self-confidence. Thus, the decline in *interest in career choice* (H3) could be an indication of a (first) defense reaction when confronted with the induced withdrawal of feelings of autonomy. Reactance occurs when adolescents feel the need to maintain their autonomy and self-determination (Brehm, 1966).

In addition, the parallel developments – concerning the decline of *feeling of autonomy* (H2) and *interests in career choice* (H3) -in both groups indicate that the questionnaire alone is thought-provoking (Bortz, 1984). The control group was also asked questions about the relevance of the social approval and the feeling of autonomy. As well as decreasing motivation to complete the questionnaires could have contributed to the poorer results in both the control and the treatment group. Another content-related factor that could also have led to a feeling of irritation and a reaction of reactance thus to the decline is the confrontation with one’s own uncertain future, especially in adolescence (Moser, 2010). The expectation that career guidance measures would relieve the students of their ‘burden’ of making independent decisions has presumably not been fulfilled. Instead, young people are confronted with both the social judgment of professions and their own uncertainty.

Nevertheless, it can be summarized that this workshop “Logic of Career Choice” encourages young people to approach their career choice more consciously. It provides space for students to reflect the need of social approval in the context of their career decisions. However, there was no measurable positive development in the sub-dimensions of career choice competence, such as the *reflection of needs* (H4) and the *intention to act* (H5) as a result of the workshop. Only *interest in career choice* (H3) and activation [*retrospectively reported career choice activities* (H6)] showed effects that were not expected in this context. *Interest in career choice* decreased significantly more in the treatment group than in the control group. Despite this, there was a significant increase in retrospectively reported career choice activities in the treatment group. According to the theory of interest (Krapp, 2002), it would be expected that a decline of *interest in career choice* would also lead to a decline in *career choice activities*. Instead, the *career choice activities reported in retrospect* indicate that this workshop had a certain activating effect. Young people who were irritated by the workshop presumably try to change this state through active engagement (“Since the last interview, I have thought a lot about my career choice”). This could mean that the participants used the time up to T3 to process new impressions, look for further information and have conversations with relevant people, which has a remarkably positive effect on the participants. A workshop like this can therefore shake up and activate, but it should necessarily be embedded in an overall career counseling program to support young people in transition.

### 6.1 Limitations

The planned intervention for the 2020/21 school year had to be postponed by a year due to the pandemic. At the beginning of data collection in the fall of 2021, recruiting schools for the study proved challenging, and pandemic-related restrictions continued to impact field conditions. Consequently, not all planned types of schools could be surveyed as originally intended. Some follow-up surveys also experienced significant delays. This article presents initial results from an ongoing research project, the data collection for which was recently completed. As typical in field studies, randomization between control and intervention classes was yet not possible, leaving the control classes without intervention during the study, but the intervention (workshop) took place after T3 (waiting control design). Although efforts were made to differentiate between control and intervention schools at the school level to minimize mutual influence; these circumstances place certain limitations on the validity of the results.

For example, limitations could arise due to some students’ limited self-awareness (e.g., due to their age), making it difficult for them to answer questions about their interests, emotions, motivations, or goals. It is essential to consider that younger students may have a limited attention span, leading to superficial responses. The questionnaire has been condensed to cover essential constructs but might still be lengthy for certain students. Pre-tests have indicated that completing the questionnaire takes approximately 20–25 min, depending on the school type.

However, it can be stated that the results have elicited a response among adolescents, prompting them to explicitly address their need for social approval. Due to the multidimensional nature of this complex phase, further data analysis is necessary. The adolescents’ stage in the career orientation process, whether they have specific aspirations or are still exploring options, likely influences their response to the workshop. Self-efficacy beliefs regarding career choice might also play a moderating role. Grade level and school type are expected to have an impact; students in upper secondary schools might face different challenges than those in secondary schools offering basic secondary education. The next steps involve refining and testing impact models. Additionally, latent class analyses could be used to identify groups with similar developmental trajectories.

Nonetheless, the present results offer intriguing insights for the further development of the concept of approval-sensitive career guidance. Furthermore, the study has emphasized the urgency of intervention studies in the field of career counseling.

## Data availability statement

The raw data supporting the conclusions of this article will be made available by the authors, without undue reservation.

## Ethics statement

Ethical approval was not required for the studies involving humans because Approval was granted by the Ministries of Education and Cultural Affairs of the Länder, as reasonableness was given on the basis of age (older than 14) and no person-sensitive data were collected. The studies were conducted in accordance with the local legislation and institutional requirements. Written informed consent to participate in this study was not required from the participants or the participants’ legal guardians/next of kin in accordance with the national legislation and the institutional requirements.

## Author contributions

SM performed the statistical analysis and wrote the manuscript. BZ and MG revised the manuscript. All authors contributed to the article and approved the submitted version.
